# Dexamethasone reduces vascular endothelial growth factor in comparison to placebo in post-operative chronic subdural hematoma samples: A target for future drug therapy?

**DOI:** 10.3389/fneur.2022.952308

**Published:** 2022-09-08

**Authors:** Ellie Edlmann, Susan Giorgi-Coll, Eric P. Thelin, Peter J. Hutchinson, Keri L. H. Carpenter

**Affiliations:** ^1^Peninsula Medical School, Faculty of Health, University of Plymouth, Plymouth, United Kingdom; ^2^Southwest Neurosurgical Centre, Derriford Hospital, Plymouth, United Kingdom; ^3^Division of Neurosurgery, Department of Clinical Neurosciences, University of Cambridge, Cambridge, United Kingdom; ^4^Department of Clinical Neuroscience, Karolinska Institutet, Stockholm, Sweden; ^5^Department of Neurology, Karolinska University Hospital, Stockholm, Sweden

**Keywords:** chronic subdural hematoma, inflammation, vascular endothelial growth factor, cytokine, trauma, head injury, dexamethasone, steroids

## Abstract

**Background:**

Chronic subdural hematoma (CSDH) is a collection of blood and fluid that arises on the brain surface due to a combination of trauma and/or inflammation. The mainstay of treatment is surgical drainage, but CSDH can recur. Dexamethasone has been shown to reduce CSDH recurrence, but its mechanism of action has not been fully elucidated. Understanding the inflammatory mediators driving CSDH formation and recurrence and how dexamethasone alters this can help develop new therapeutic strategies.

**Methods:**

A subgroup of adult patients recruited to the Dex-CSDH trial, randomized to dexamethasone or placebo, who had surgery for their CSDH, were included. CSDH fluid and peripheral blood were collected intraoperatively, from post-operative drains and operated recurrences. Samples were analyzed using a 12-plex panel of inflammatory mediators. Clinical patient data were also reviewed.

**Results:**

A total of 52 patients, with a mean age of 76 years, were included. Five recurrent CSDHs occurred. Vascular endothelial growth factor (VEGF) had the highest concentration across all CSDHs, and only matrix metalloproteinase (MMP)-9 had lower concentrations in CSDH compared to plasma but was increased in recurrent CSDHs. The interleukin (IL)-10 concentration was significantly lower in primary CSDHs that recurred. Most inflammatory mediators increased post-operatively, and dexamethasone significantly reduced the post-operative peak in VEGF on day 2, compared to placebo.

**Conclusion:**

It is evident that VEGF plays a critical role in the inflammatory response in CSDH. The post-operative reduction with dexamethasone could signal the mechanism by which it reduces recurrence. Novel therapies with a better side-effect profile than dexamethasone should be targeted at VEGF or potential alternatives such as IL-10 supplementation.

## Introduction

Chronic subdural hematoma (CSDH) is a collection of blood and fluid overlying the brain, encapsulated within a membrane. It primarily affects older patients and has long been considered a consequence of traumatic head injury causing bleeding into the subdural space, although it can occur in the absence of a history of head injury (1–3). It is primarily treated with surgical drainage but can recur, resulting in significant morbidity (4). In recent years, there is a growing interest in the role of inflammation in CSDH formation (5, 6). Local elevation of many inflammatory mediators such as chemokines, cytokines, and growth factors within CSDH has been reported (7–17). These mediators are produced by cells within the CSDH fluid and/or by the endothelial cells within the CSDH membrane (7, 9, 11, 18). The proliferation of the CSDH membrane, with its abundance of fibroblasts, inflammatory cells, and highly permeable capillaries, is believed to lead to the continued migration of blood, fluid, and cells into the CSDH cavity, accumulating over time (7, 8, 11, 19–21).

Pharmacological treatments targeted at modifying inflammation are the most common focus of CSDH trials (22). The steroid medication, dexamethasone, has been demonstrated to successfully aid CSDH resolution and reduce recurrence (23, 24). The precise mechanism by which this occurs has never been investigated, although it is anticipated to be related to dexamethasone's anti-inflammatory effects (25–27). Despite successfully reducing recurrence, patients with CSDH treated with dexamethasone had worse clinical outcomes in a randomized trial of dexamethasone vs. placebo (Dex-CSDH trial) (24). This was likely due to the side-effect profile of dexamethasone, particularly increased systemic infections. This enforces the need for similar mechanistic drugs but with fewer side effects. To understand the pathological inflammatory mechanisms driving CSDH and why dexamethasone was successful in reducing recurrence, a sub-study with inflammatory analysis of CSDH fluid from patients participating in the main Dex-CSDH trial was undertaken (28).

Intraoperative CSDH fluid analysis has been performed in the past (7–9, 11, 14–16), but never in the context of pharmacological treatment, and very less regarding post-operative changes. This study aimed to compare inflammatory mediator concentrations between intraoperative CSDH fluid and plasma samples, with the hypothesis that inflammatory mediators would be higher in CSDH fluid vs. plasma, higher in recurrent vs. primary CSDH, and reduced in post-operative drain fluid samples over time following surgical washout, different between sides of a bilateral CSDH, and finally lower in patients treated with dexamethasone, potentially as a mechanism for reducing CSDH recurrence. Novel markers, not previously investigated in CSDH but shown to be elevated in brain injury experiments, were also investigated, including IL-1α, macrophage inflammatory proteins (MIP)-1α, and MIP-1β (29, 30).

## Materials and methods

### Patients

Patients consented to inclusion in this sub-study either individually, by a next-of-kin, or by an independent healthcare professional as part of the main Dex-CSDH trial (24). This was prospectively approved by a research and ethics committee (15/NW/0171). The inclusion criteria stated that all patients were adults with a symptomatic CSDH who had been admitted to a neurosurgical unit within the past 72 h. Patients were excluded if they had steroid treatment in the last month, a contraindication to steroids, a cerebrospinal shunt, intolerance to lactose, gelatin, or any other of the drug excipients, a history of psychosis, active malignancy, or were receiving immunosuppressive drug therapy. Patients were recruited consecutively from a single site participating in the Dex-CSDH trial once ethical approval had been obtained for the sub-study. Patients were only eligible for the sub-study if they were recruited prior to the surgery taking place.

All patients had surgical drainage of their CSDH with paired intraoperative blood and CSDH fluid samples collected. In the case of bilateral CSDH, separate CSDH samples were collected from each side. Patients with a routine post-operative subdural drain left *in situ* had CSDH drainage samples collected at regular intervals after surgery until drain removal.

All patients were randomized to dexamethasone or placebo in addition to surgery as part of the Dex-CSDH trial. Patients allocated to the dexamethasone arm, but who did not receive the drug preoperatively (some were randomized post-operatively), were grouped with the placebo patients for analysis of intraoperative samples. Clinical data were collected on all patients regarding demographics, history of trauma, and anti-thrombotic use. To investigate the impact of age on the inflammatory profile, patients aged under 75 years were compared to those aged 75 years and over, a cut-off commonly used for “older adults” in the clinical literature. Outcome data at 3 and 6 months were collected as a modified Rankin Score (mRS), which is a scale from 0 (no symptoms) to 6 (death). Outcomes were dichotomised into favorable (mRS 0–3) and unfavorable (mRS 4–6), where 3 corresponds to moderate disability (*able* to walk unassisted) and 4 means moderately severe disability (*unable* to walk unassisted).

Intraoperative samples were grouped into the primary and recurrent CSDH samples. The recurrent samples were also linked to their “paired” primary samples from the same patient. In the case of multiple recurrences, each recurrence was paired with the first CSDH. The primary samples from the patients who went on to have a recurrence were also compared to the primary samples in patients who had no recurrence.

### Sampling protocol and analysis

Intraoperative blood samples were collected by the anesthetist, and 10 ml of CSDH fluid was aspirated with a syringe prior to irrigation. Samples were immediately dispensed into ethylenediamine tetra-acetic acid tubes. Post-operative samples were collected daily from subdural drainage bags from 8 h post-operatively for a maximum of 72 h (when all drains were removed) with complete emptying of the bag between sampling. Samples were stored at 4°C until centrifuged, and then the supernatant was stored at −75 °C prior to analysis. Samples were thawed and analyzed using a magnetic bead-based immunoassay on a Luminex 200 analyser (Luminex Corporation, Austin, TX, USA) with custom 12-plex ProcartaPlex human cytokine and chemokine assay kits (Affymetrix eBioscience, Thermo Fisher Scientific, Paisley, UK) for markers in Table 1. All standards and samples were analyzed in duplicate.

**Table 1:** Panel of inflammatory mediators analyzed.

Cytokines	Interleukins (IL); IL-1α, IL-1β, IL- 6, IL- 8, IL-10 Tumor Necrosis Factor; TNF-α
Chemokines	Macrophage Inflammatory Proteins; MIP-1α, MIP-1β Monocyte Chemoattractant Protein; MCP-1 Interferon-gamma-induced Protein; IP-10
Other inflammatory molecules	Vascular Endothelial Growth Factor; VEGF Matrix-Metalloproteinase; MMP-9

### Statistical analysis

Statistical analysis was performed using GraphPad Prism 7 (GraphPad Software, La Jolla, CA, USA). Data were found to be not normally distributed. Paired samples were therefore analyzed using the Wilcoxon matched-pairs signed rank test and unpaired samples with the Mann-Whitney test. A Spearman's rank correlation coefficient (Spearman's rho) was used to display the relationship between any two mediators. Post-operative samples were compared as percentage change across each time point compared to the intraoperative value, and statistical analyses were done using an unpaired *t*-test as these data were normally distributed. All statistical analyses assumed a significance level of *p* < 0.05.

## Results

A total of 100 patients were recruited to the main Dex-CSDH trial at the lead site within the study time window, with 51 patients recruited prior to surgical intervention and thus eligible for inclusion in this study. Samples were collected from 56 operations, including five recurrences in four patients. This led to 67 separate CSDH samples (61 primary and six recurrent), due to the sampling of bilateral CSDH in 11 patients. The mean and median patient age was 76 years and 73% of patients were men. There were no significant differences in any inflammatory mediator in primary intraoperative CSDH samples between patients aged under 75 years and over 74 years (Supplementary Table S1).

### Summary of the intraoperative inflammatory profile

**Figure 1:**
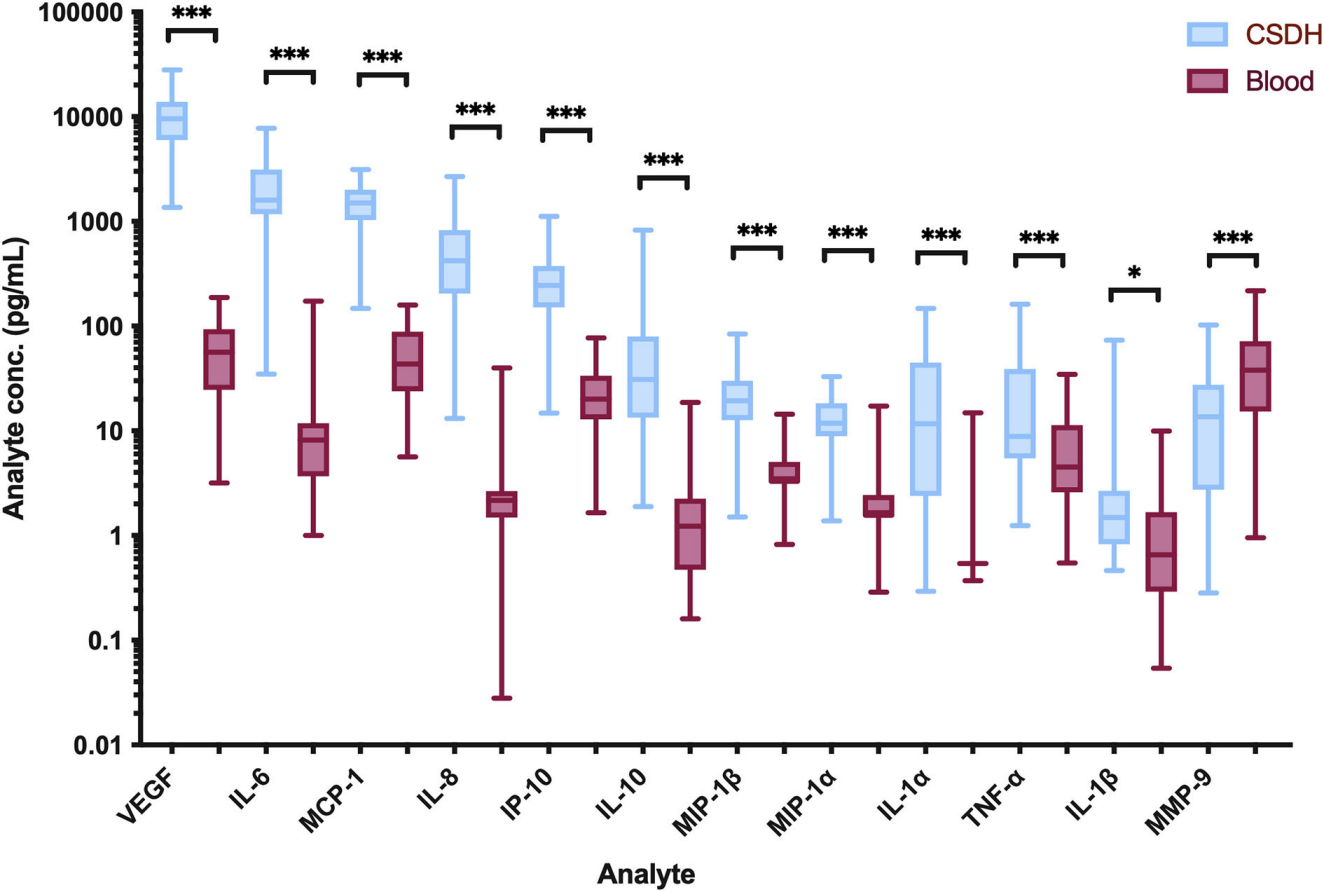
Intraoperative inflammatory marker concentrations detected in CSDH fluid (blue) and blood plasma (red). All values are the mean of two replicates, *n* = 67 from 51 patients. Line (median), box (interquartile range), and whiskers (minimum-maximum). Statistically significant differences are shown as *p < 0.05 and ***p < 0.0001.

**Figure 2:**
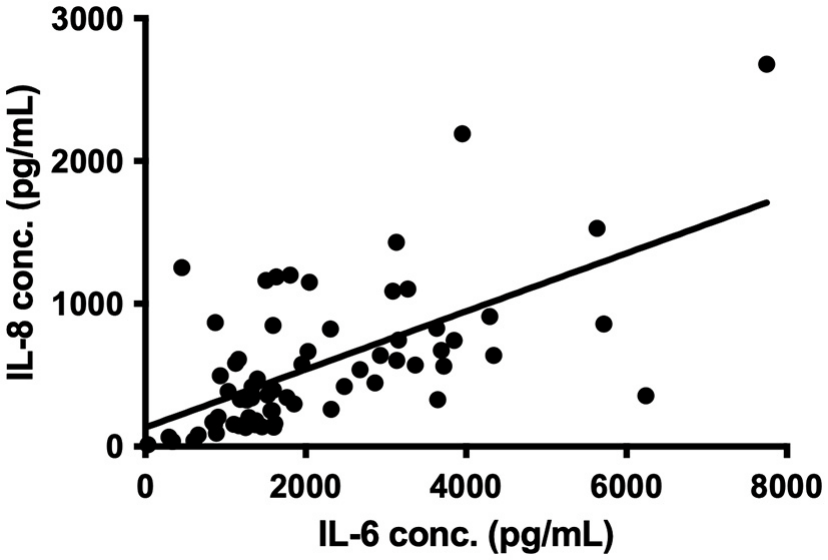
Correlation between IL-6 and IL-8 concentration (conc.) in 67 CSDH fluid samples, R = 0.615, *p* < 0.0001 on Spearman correlation, line represents linear regression (R^2^ = 0.381).

All the inflammatory mediators, apart from MMP-9, were significantly raised in the CSDH samples compared to plasma samples (Figure 1). The vascular endothelial growth factor (VEGF) had the highest median concentration in CSDH fluid (9,485 pg/ml) and the largest difference in comparison to plasma (approximately 170 times higher). Other inflammatory mediators with particularly high concentrations were IL-6, MCP-1, IL-8, and IP-10. The only mediator with a significantly lower concentration in the CSDH fluid compared to plasma was MMP-9. The highest correlation of markers was observed between IL-6 and IL-8 (R = 0.615, *p* < 0.001, Figure 2). Other moderately significant correlations were VEGF with IL-6 and MIP-1β, and IL-1α with IL-8 and TNF-α. The 10 bilateral CSDHs sampled showed a good correlation of mediator concentrations between sides (Supplementary Table S2).

### Primary and recurrent CSDH samples

**Figure 3:**
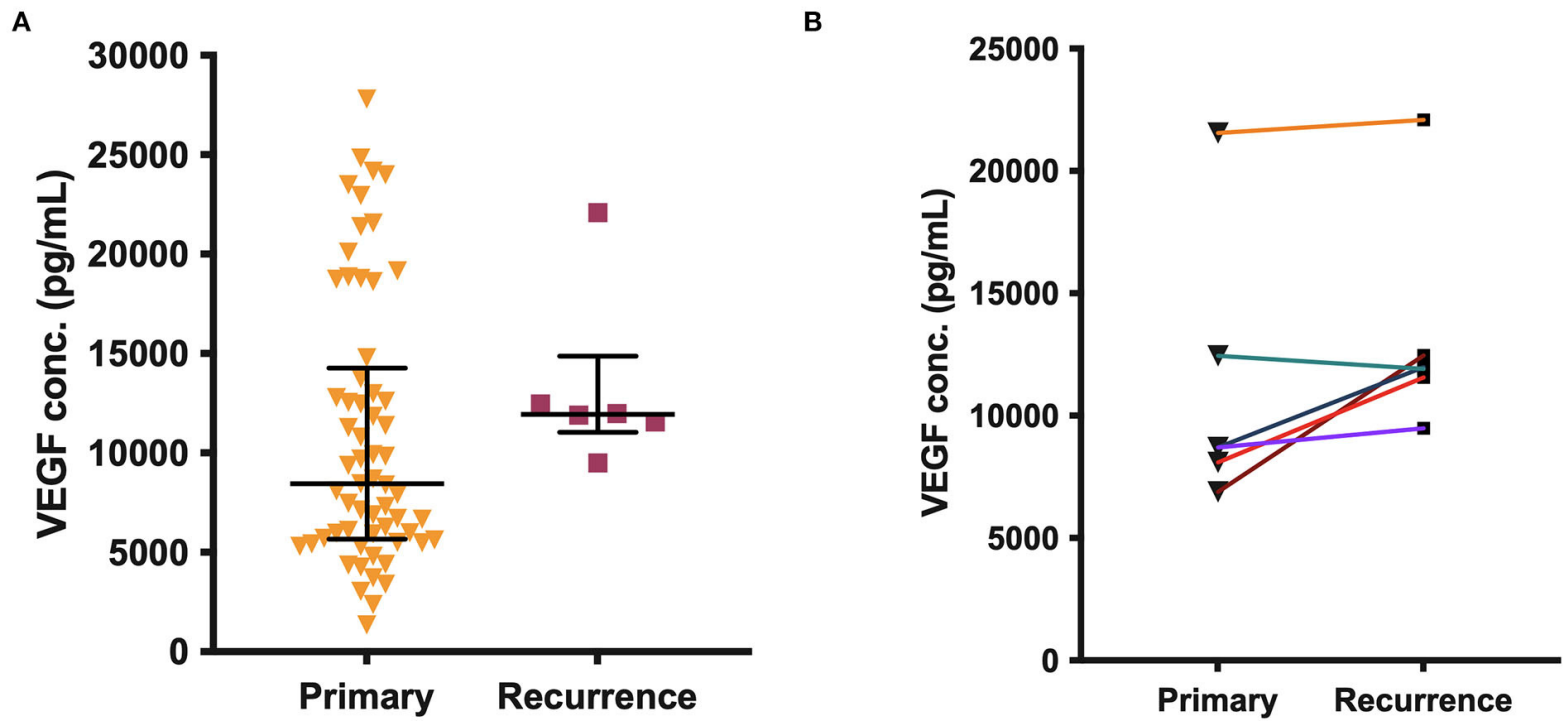
(**A)** VEGF concentration in all primary (*n* = 61) and recurrent (*n* = 6) CSDH fluid samples, line (median), and bars (IQR). **(B)** Paired primary-recurrent CSDH samples (*n* = 6), where n is the number of samples. No significant differences are shown.

There was no significant difference in VEGF concentrations between all primary CSDHs (median 8,443 pg/ml) and recurrent CSDHs (median 11,947 pg/ml, *p* = 0.161), or between the paired primary-recurrence samples (*p* = 0.094, Figure 3). In the paired samples, the VEGF level was either similar to, or higher in the recurrent samples, with a mean percentage increase of 28%.

**Figure 4:**
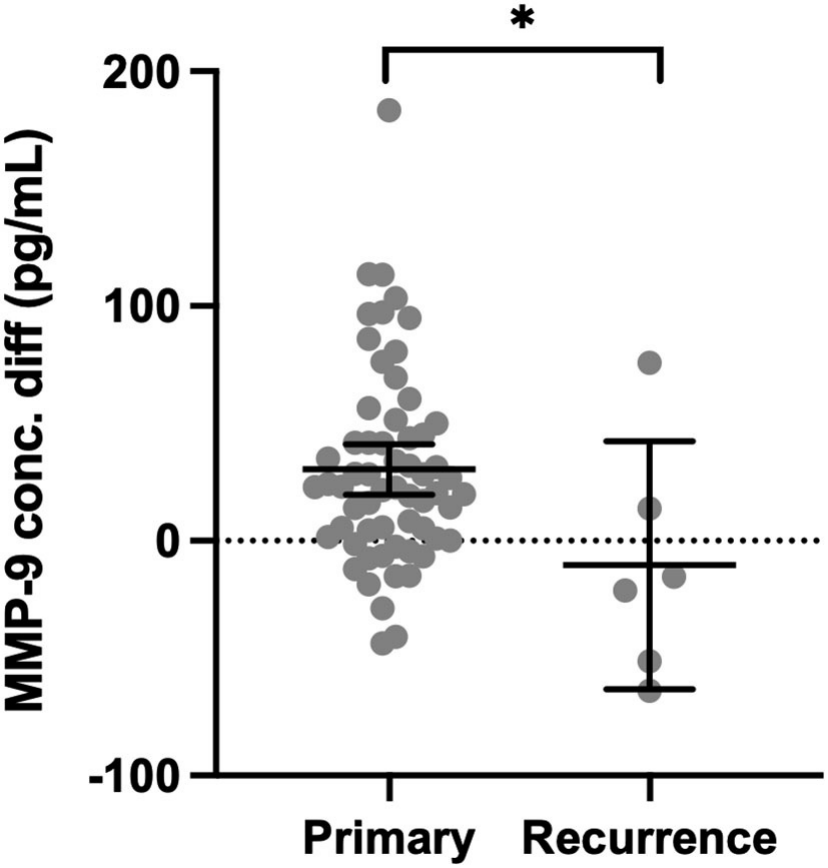
Concentration difference (conc. diff) for MMP-9 in primary CSDH (*n* = 61) and recurrent CSDH (*n* = 6) fluid, as determined by plasma concentration minus CSDH fluid concentration. Line (median), bars (IQR), and statistically significant differences are shown as *p < 0.05.

Matrix metalloproteinase-9 was higher in CSDH fluid compared with plasma in only 13/61 (21%) primary CSDH samples but increased to 4/6 (67%) recurrent CSDH samples. The mean concentration difference (plasma minus CSDH concentration) was significantly lower in recurrent samples (*p* = 0.020, Figure 4).

**Figure 5:**
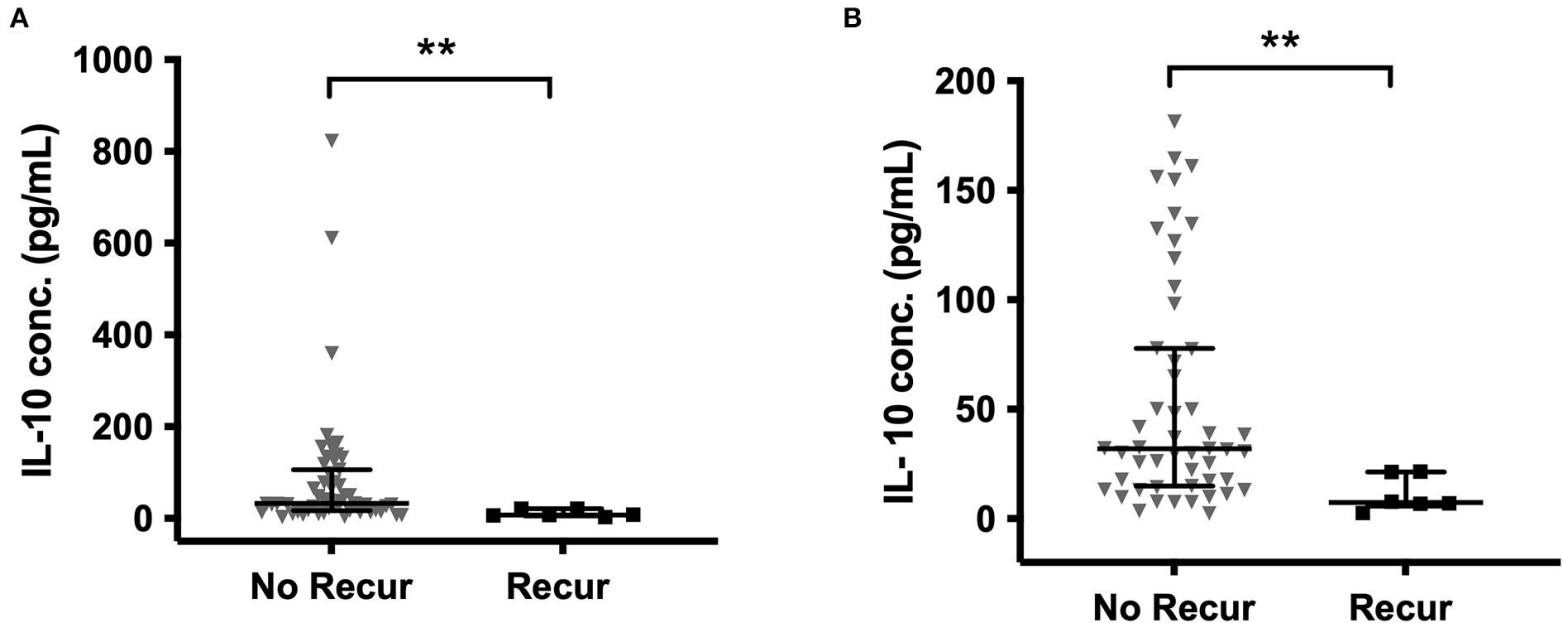
IL-10 in primary CSDH fluid samples that went on to recur and those that did not recur **(A)** all data **(B)** outliers excluded (excluded by interquartile range × 1.5 rule). No recurrence (No Recur) *n* = 55; Recurrence *n* = 6. Line (median), bars (IQR), and statistically significant differences are denoted as p ≤ 0.005 (**).

When comparing all primary and recurrent samples, significantly higher concentrations of IL-6 (*p* = 0.004), TNF-α (*p* = 0.035), and IL-1β (*p* = 0.012) were found at recurrence (Supplementary Figure S1). In the paired primary-recurrent samples, IL-8, TNF-α, and IL-1α were all significantly increased at recurrence (*p* = 0.031). All other mediators, apart from MCP-1, had a positive mean percentage difference at recurrence compared to primary, despite not reaching significance. IL-10 was significantly higher in primary CSDHs that did not go on to recur compared to those that did recur (*p* = 0.002), and this remained with the removal of outliers (Figure 5).

### Post-operative CSDH drain samples

**Figure 6:**
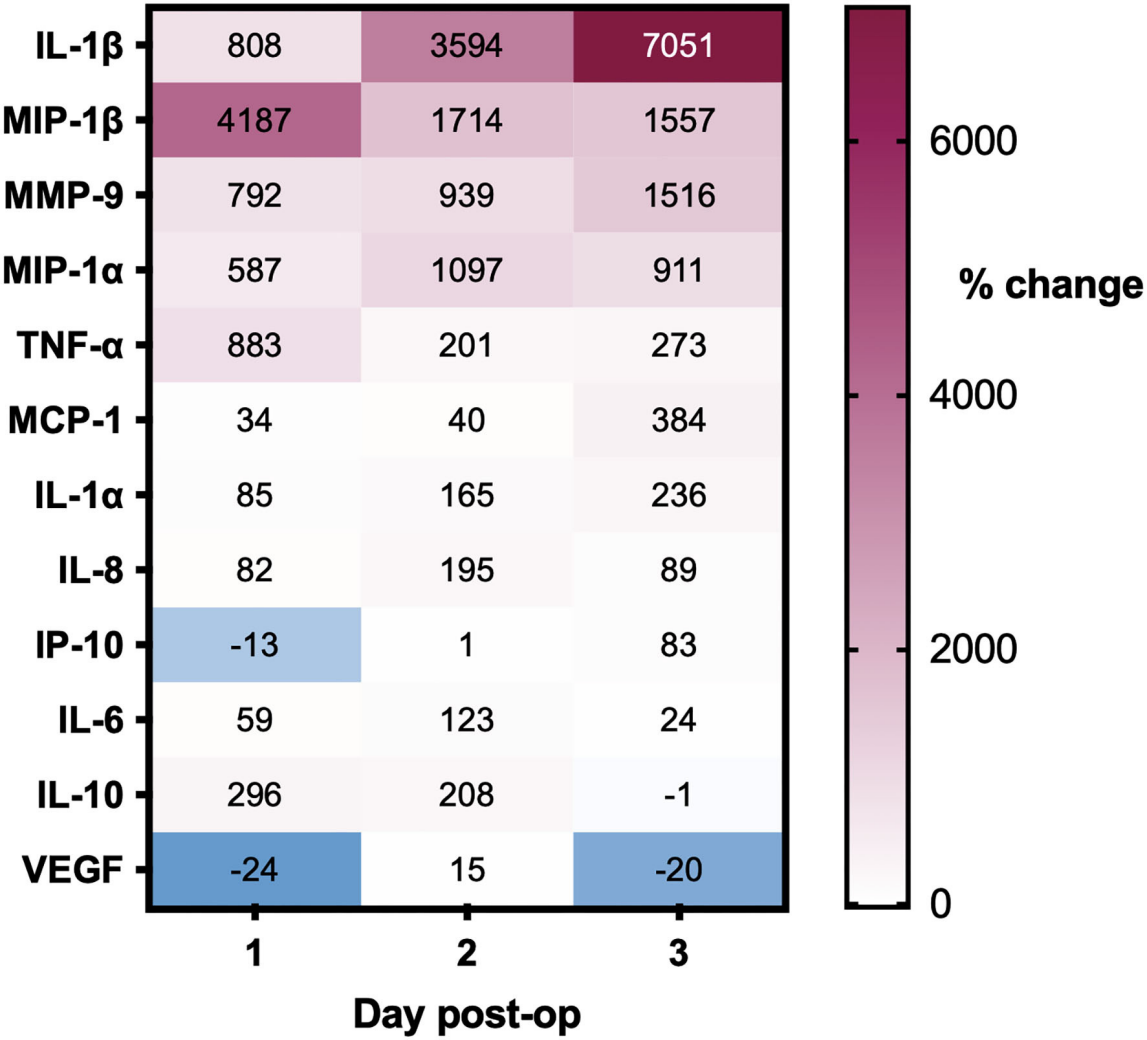
Percentage changes in CSDH drain analyte concentrations over 3 days post-operatively, all compared to intraoperative CSDH fluid concentrations. Day 1 = 28 samples, day 2 = 10 samples, and day 3 = 4 samples (see full data with standard deviation in Supplementary Table S1).

Post-operative drain samples were collected from 28 CSDHs in 25 patients (three bilateral), with a mean age of 77 years. All drains were sampled on the first day post-operatively, 10 on days 2 and 4, and day 3. The summary of percentage change (compared to intraoperative samples) across days for all mediators is displayed in Figure 6. The majority of mediators showed a positive percentage change across successive time points. Only VEGF showed a large decrease in concentration on day 1 (average−24%), which increased significantly on day 2 (+15%) and lowered again in the few samples on day 3 (−20%). In relation to recurrence, only MCP-1 was significantly higher on a post-operative day 1 in patients who went on to have a recurrence (n=4) compared to those who did not (*n* = 22, *p* = 0.009). There were insufficient samples on days 2 and 3 to assess the relationship to recurrence.

### Dexamethasone vs. placebo

Of the 51 patients sampled, 22 were randomized to dexamethasone and 29 to placebo. Only 11 dexamethasone patients received it preoperatively (excluding one who completed the course 44 days prior), making 40 intraoperative patients “no dexamethasone” (No-Dex) and 11 dexamethasone (Dex). Recurrence occurred in 1 Dex patient compared to 4 No-Dex patients (Table 2). There were 12 Dex samples (one bilateral) and 49 No-Dex. Significantly higher intraoperative concentrations of IL-6 (*p* = 0.018), IL-8 (*p* = 0.011), MIP-1β (*p* = 0.038), TNF-α (*p* = 0.022), and IL-1α (*p* = 0.023) were seen in Dex compared to No-Dex samples (Supplementary Figure S2). No difference in concentration was observed for VEGF or the remaining markers. Analysis of the cumulative preoperative dose of Dex showed a continuous rise in inflammatory mediator concentration with increasing doses of Dex for VEGF, MMP-9, and IL-6 (Supplementary Figure S3). All other mediators increased in concentration with Dex doses of 8–48 mg, then decreased at the highest dose (54–72 mg, equivalent to 4 or 5 days of treatment).

**Table 2:** Treatment groups and chronic subdural recurrence (Dex = dexamethasone).

**No. of patients**	**Treatment group: dose received pre-op**	**Recurrence: no. of days after 1st operation**
29	PLACEBO	4 in 3 patients (1 bilateral); day 11, 13 and 14.
11	Dex; 0 mg (started post-op)	2 in 1 patient; day 41 & 50
2	Dex 8 mg	0
2	Dex 16 mg	0
1	Dex 32 mg	1 patient; day 14
1	Dex 40 mg	0
1	Dex 48 mg	0
1	Dex 54 mg	0
1	Dex 60 mg	0
1	Dex 72 mg	0
1	Dex 124 mg (full course)	0

**Figure 7:**
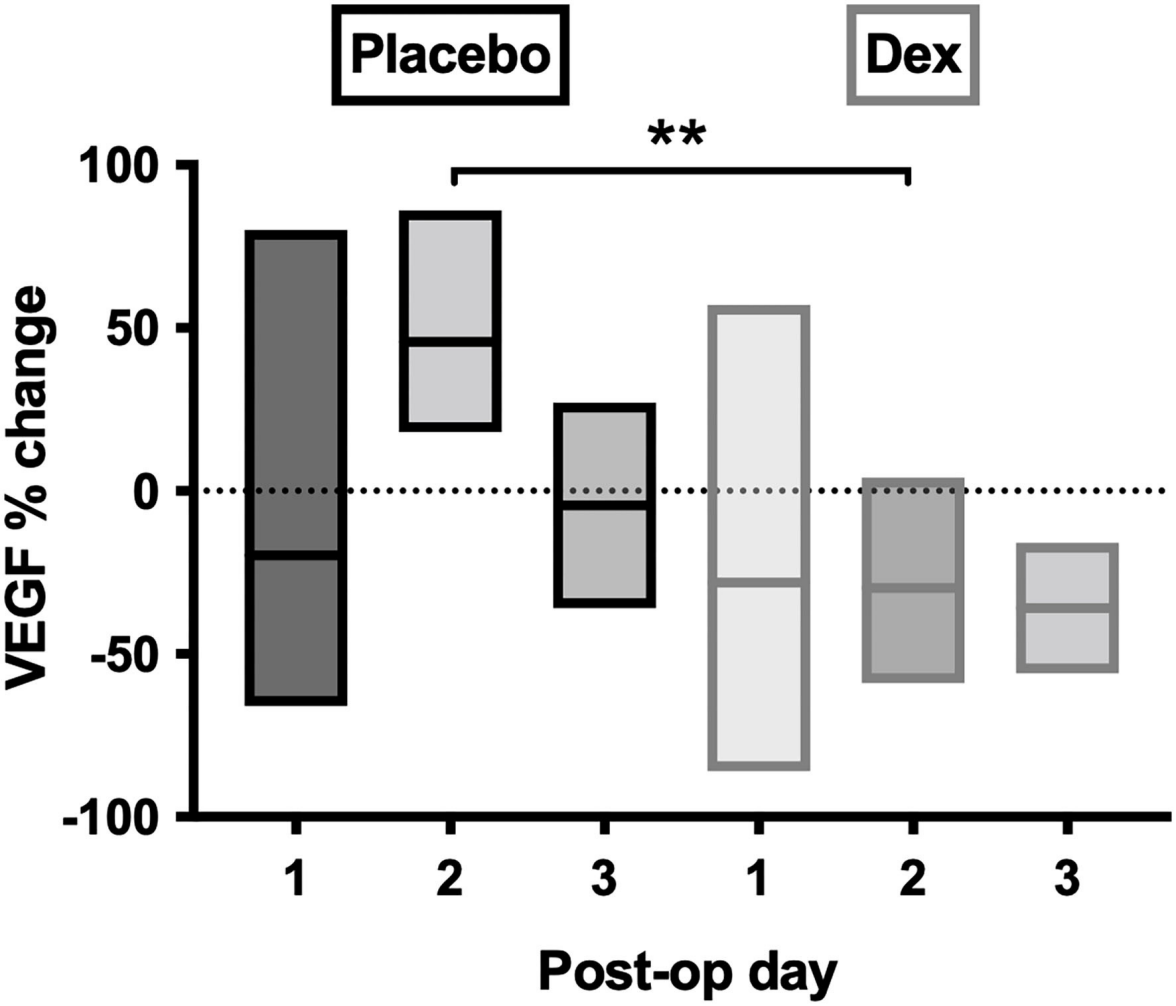
Percentage-change of VEGF concentrations in post-operative CSDH drain samples by day. Day 1 = 28 samples, day 2 = 10 samples, and day 3 = 4 samples. Line (mean) and box (range). Statistically significant differences are denoted as *p* ≤ 0.005 (**).

Post-operative samples showed a day-2 VEGF peak in the placebo group, which was significantly reduced in the Dex group (*p* = 0.004, Figure 7). A similar, but non-significant, the reduction was seen in the Dex group on a post-operative day 2 for IL-6, IL-8, IL-10, IL-1α, IL-1β, MIP-1α, and MIP-1β.

### Clinical correlations

**Figure 8:**
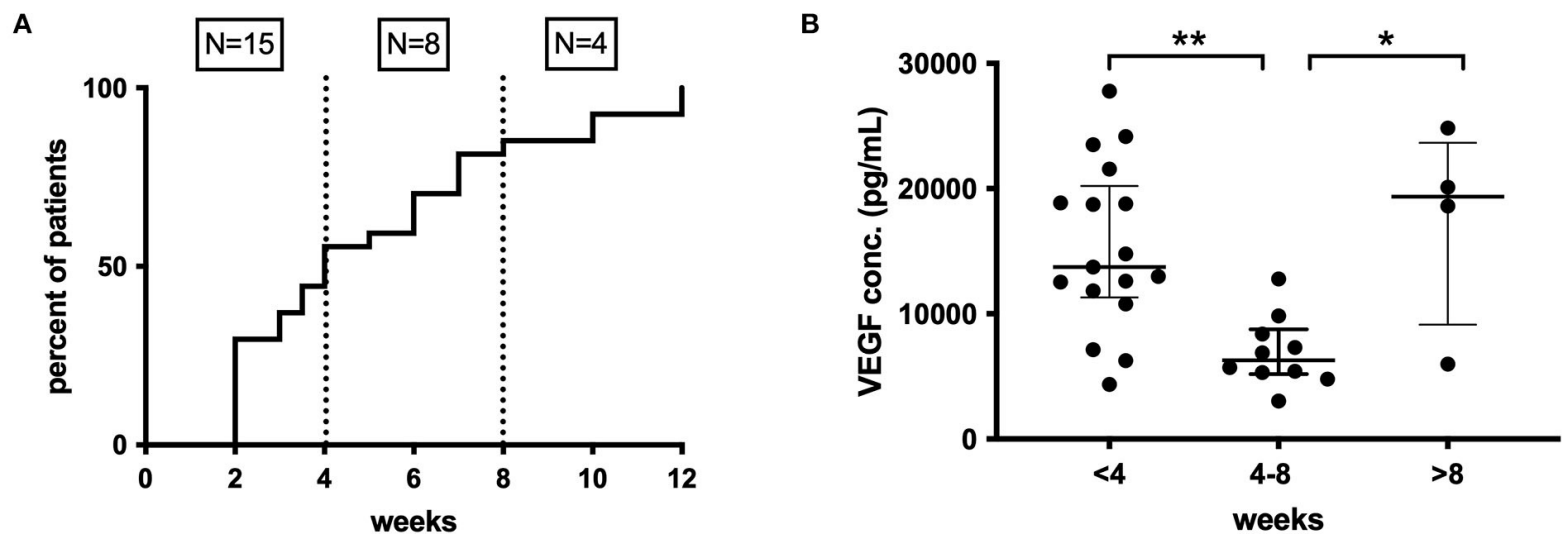
**(A)** Time course of CSDH diagnosis following trauma, <4 weeks (*n* = 15), 4–8 weeks (n = 8), and after 8 weeks (*n* =4). **(B)** Pattern of intraoperative CSDH fluid VEGF concentrations at different time intervals post-trauma. Line (median) and bar (IQR). Statistically significant differences are denoted as *p* ≤ 0.005 (**) or *p* ≤ 0.05 (*).

Preoperative anti-platelet (21% of patients) and oral anticoagulant (36% of patients) therapy had no impact on inflammatory mediator profiles. A patient-reported history of recent head trauma was present in 30/51 (61%) patients, and only IL-1α (*p* = 0.025) and TNF-α (*p* = 0.026) showed significantly higher inflammatory mediator concentrations in these patients. The time interval between trauma and CSDH diagnosis was known for 27/30 patients and was 2–12 weeks (median 4). When grouped into three equal time periods, a significant difference in the concentration of VEGF was observed between <4 weeks and 4–8 weeks (*p* < 0.001) and between 4–8 weeks and >8 weeks (*p* = 0.036) (Figure 8). No significant changes were found for any other markers.

## Discussion

This study highlights the local over-activation of inflammatory mediators within CSDH, in particular VEGF, and that the response to dexamethasone is complex but may be most relevant in the early post-operative period.

### Intraoperative analysis and recurrence

All mediators, apart from MMP-9, were significantly elevated in CSDH fluid compared to plasma, supporting the established theory of a localized inflammatory response. Novel markers, such as IL-1α, MIP-1α, and MIP-1β, are also now indicated in this process. Bilateral CSDHs have a relatively congruous response between sides, and there may be movement of inflammatory cells across the midline dura, which harbors its own source of inflammatory cells and vascular supply (31). The highest concentration mediator in CSDH fluid was VEGF, as identified in previous studies, and may be the primary driver of CSDH formation (7, 9, 11, 12, 16). It has a key role in angiogenesis and vascular permeability, both critical to CSDH propagation (7, 32). While VEGF concentration did not increase significantly at recurrence in our study, corroborated by previous research (11), there are still on-going high levels suggesting an active role. Some patients with very high VEGF concentrations at primary surgery do not develop a recurrence. It may be that adequate surgical washout is sufficient to switch off the drive for CSDH formation, as supported by reduced recurrence risk with smaller residual volumes of CSDH on post-operative imaging (33).

Cytokines can be considered to have primarily pro- or anti-inflammatory actions and are recognized to work in cascades, with the balance between them potentially relevant to CSDH recurrence (13). We observed high concentrations of IL-6 and IL-8, which were moderately correlated and have recognized pro-inflammatory actions (34, 35). Importantly, IL-10, often considered an anti-inflammatory cytokine, was the only marker where significantly lower levels were observed in primary CSDHs that went on to recur compared to those that did not. This suggests that patients with low levels of IL-10 may lack some of the “anti-inflammatory” balance required to promote CSDH resolution and avoid recurrence. The largest shift in activity between primary and recurrent CSDH was seen with MMP-9, essential for angiogenesis and inflammation and likely to play a role in leaky capillary formation in CSDH membranes (10, 36–38). There were higher concentrations of plasma in 85% of primary CSDHs, which reversed to higher concentrations in CSDH fluid for 67% of recurrent CSDHs. The recurrence numbers are small, but this data may indicate a role for MMP-9 reactivation in CSDH recurrence, allowing new membranes and further hemorrhage. A sustained increase in MMP-9 was also observed in the post-operative drain samples (Figure 6).

### Post-operative CSDH drain samples

We hypothesized that inflammatory mediators would be reduced post-operatively due to irrigation of the collection; however, largely the converse occurred. Renewed inflammatory response to surgery is the likely cause, and one previous report suggests raised inflammatory mediators attract neutrophils and macrophages and mediate healing post-operatively (39). VEGF is the only mediator that showed a marked percentage decrease on day 1 post-operatively, which may reflect a washout of very high concentrations which cannot be rapidly replaced but does increase again by day 2. Day 3 samples were collected from too few patients to be informative. Only elevated MCP-1 concentrations in post-operative drain samples signified a higher likelihood of CSDH recurrence, suggesting that the post-operative concentration of mediators alone does not help predict recurrence.

### The effect of dexamethasone

It was anticipated that preoperative dexamethasone would reduce inflammatory mediator concentrations intraoperatively and post-operatively. Most patients had 1 to 3 days of preoperative dexamethasone, and it has a rapid onset, reaching peak plasma concentration within 1–1.5 h with a biological half-life of 36–54 h (25, 40–42). Nonetheless, there are no studies assessing drug penetration into a CSDH, which despite its vascular membrane only accumulates blood and/or fluid extravasation slowly over weeks. It is difficult to know to what extent, and how quickly, dexamethasone can infiltrate the CSDH fluid and influence the inflammatory profile within. Several markers (IL-6, IL-8, MIP-1β, TNF-α, and IL-1α) were significantly increased in the patients who were given preoperative dexamethasone, and some showed incremental increases with higher dexamethasone dosing preoperatively, the opposite of what was anticipated. Several mediators were reduced after several days of treatment, therefore, the anti-inflammatory effect may take time to infiltrate the subdural collection. The post-operative drain samples showed a general reduction in the increase of most inflammatory markers with dexamethasone compared to placebo, which was more in-keeping with the expected anti-inflammatory response. This likely relates to the fact that the dexamethasone can target the inflammatory markers being produced acutely post-operatively in response to surgery, while the large pre-existing pool has been drained. A significant change was observed for VEGF, which no longer showed post-operative peak concentrations on day 2, but was instead leveled. Despite the small numbers, there is an indication that the anti-inflammatory role of dexamethasone allows a “dampening down” of the post-operative inflammatory response, particularly VEGF. This may help explain the mechanism behind the role of dexamethasone in reducing CSDH recurrence (from 7.1 to 1.7%), found in the large randomized trial these patients were sampled from (24). While we do not know what occurs in the inflammatory profile on the following days, due to routine practice of drain removal at 48 h, it is understood that CSDHs take weeks to accumulate, and therefore, even these early differences may determine the chance of a CSDH-recurrence-cycle initiating.

### Does trauma initiate a cyclical inflammatory response?

The history of trauma appeared to have less impact on the inflammatory profiles observed. This is limited by possible recall bias, as patients may forget or fail to register minor trauma, particularly if it was a long time previously. There are also challenges in interpreting the data in time-from-trauma, as many patients with CSDH are “recurrent” fallers, and therefore, although they may report a recent trauma, it is uncertain whether an earlier trauma was responsible for initiating the CSDH. Despite this, there does appear to be a significant increase in concentrations of VEGF, in particular in patients presenting within 4 weeks of trauma, compared to 4–8 weeks, which is more varied beyond 8 weeks (Figure 8). This would support the hypothesis of an early pro-inflammatory state which then becomes more dormant and/or cyclical over time.

Data on anti-thrombotics suggested no change in the inflammatory profile, despite aspirin and clopidogrel being shown to have anti-inflammatory properties (43–45). These data are confounded by the variation in time from stopping anti-thrombotics to surgery.

### Limitations

This study was a sub-study within a larger trial and recruited a smaller number of patients as it started later, was only recruiting from the lead site, and could only include patients who consented prior to surgery. This led to a smaller sample size than the main trial and is therefore not powered to make conclusions regarding the CSDH recurrence rate. However, the study was intended as a mechanistic study to shed light on the role of dexamethasone (28). Given that the final results of the larger trial have found dexamethasone to result in worse outcomes for patients, data on patients treated with dexamethasone are unlikely to be available again, and these data can still be used to extrapolate from for future drug trials.

### Future treatment strategies

Understanding and identifying key mediators may help develop non-surgical therapeutic interventions for CSDH management. It is evident that VEGF is a significant factor in CSDH formation and is likely to be relevant in CSDH recurrence. The effect of dexamethasone in reducing the day-2 post-operative peak concentrations of VEGF is an insight into the important early post-operative anti-inflammatory effects needed to aid CSDH resolution. The success of dexamethasone in reducing recurrence but with an unacceptable side-effect profile may indicate the need for a similar but more-targeted drug (24). VEGF has been implicated in neovascularisation in other conditions, such as diabetic retinopathy, which is characterized by a pre-retinal neovascular membrane not dissimilar to the CSDH neomembranes, with a network of highly permeable vessels surrounded by fibroblasts and macrophages (46). Patients with proliferative retinopathy have higher circulating plasma VEGF, which has shown trends of decline following laser treatment and resolution of neovascularisation (47). Anti-VEGF treatments are a potential future consideration for CSDH treatment, which may target similar anti-inflammatory mechanisms as dexamethasone but avoid the high-risk side effects. Novel drugs targeting MMP-9 are also in development and may offer another target to the angiogenic processes, which appear to be at play, particularly apparent in the recurrent CSDH samples (48). Finally, the significantly lower levels of IL-10 in patients that go on to have recurrent CSDH may indicate a role for IL-10 supplementation as a therapy. Inflammatory conditions, such as inflammatory bowel disease, have focused on IL-10 as a treatment but are yet to see clinical improvements due to challenges with drug stability, bioavailability, and the complexity of immune responses (49). However, newly engineered models of IL-10 may improve the potential to develop IL-10 therapy further and apply it more successfully as a treatment (50). This may be a future avenue of interest in patients with CSDH.

## Conclusion

There is clear evidence of a pro-inflammatory environment in CSDH, and the primary impact of dexamethasone may be a reduction in the post-operative inflammatory mediator spike. Targeted anti-VEGF therapy is a good place to start for future drug trials in CSDH, with alternative tentative drug targets also identified.

## Data availability statement

The datasets presented in this study can be found in online repositories. The name of the repository and accession number can be found below: G-Node Infrastructure (GIN) repository, https://gin.g-node.org/eedlmann/CSDH-cytokine.

## Ethics statement

The studies involving human participants were reviewed and approved by Northwest Haydock Park REC, United Kingdom, 15/NW/0171. The patients/participants provided their written informed consent to participate in this study.

## Author contributions

EE: conceptualization, data curation (lead), formal analysis, investigation (lead), methodology, administration, and writing the original draft. SG-C: conceptualization, data curation, investigation, methodology, writing—reviewing, and editing (equal). ET: data curation, writing—reviewing, and editing (equal). PH: conceptualization, methodology, supervision, writing—reviewing, and editing (equal). KC: conceptualization, methodology, supervision, writing—reviewing, and editing (lead). All authors contributed to the article and approved the submitted version.

## Funding

The Dex-CSDH trial was funded by the National Institute for Health Research HTA grant (13/15/02). The Luminex 200 analyser was purchased with MRC funding (Grant No. G0600986 ID79068). EE—A Royal College of Surgeons of England Fellowship, funded by the Rosetrees Trust; PH—National Institute for Health and care Research (Professorship), Biomedical Research Center, Brain Injury MedTech Co-operative, Senior Investigator Award, and the Royal College of Surgeons of England; KC—National Institute for Health and care Research Biomedical Research Center, Cambridge (Neuroscience Theme; Brain Injury and Repair Theme); SG-C—National Institute for Health and care Research Biomedical Research Center, Cambridge (Neuroscience Theme; Brain Injury and Repair Theme); ET—Swedish Brain Foundation (Hjärnfonden, Mattsons Stiftelse #FO2019-0006), Swedish Medical Society (#SLS-587221) and the Swedish Society for Medical Research (SSMF).

## Conflict of interest

The authors declare that the research was conducted in the absence of any commercial or financial relationships that could be construed as a potential conflict of interest.

## Publisher's note

All claims expressed in this article are solely those of the authors and do not necessarily represent those of their affiliated organizations, or those of the publisher, the editors and the reviewers. Any product that may be evaluated in this article, or claim that may be made by its manufacturer, is not guaranteed or endorsed by the publisher.
