# Supplementary material for: Dexamethasone reduces vascular endothelial growth factor in comparison to placebo in post-operative chronic subdural hematoma samples: A target for future drug therapy?
Source: Front Neurol. 2022 Sep 8;13:952308. doi: 10.3389/fneur.2022.952308 (PMC9492873; doi:10.3389/fneur.2022.952308)
Supplement: Supplementary file 1 [file Data_Sheet_1.pdf]

Dexamethasone reduces vascular endothelial growth factor in comparison to placebo in post-operative chronic subdural hematoma samples; a target for future drug therapy?

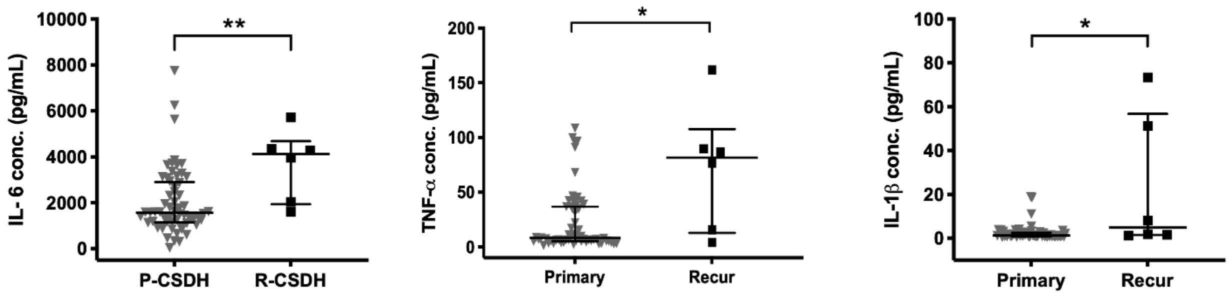

Figure S1; intra-operative inflammatory mediator concentrations (conc.) in primary (P) CSDH (n=61) compared to Recurrent (R/Recur) CSDH (n=6) for **(A)** IL-6, **(B)** TNF-α, **(C)** IL-1β. Line (median), bars (IQR), statistically significant differences denote as $P \leq 0.05$ (*), $P \leq 0.005$ (**)

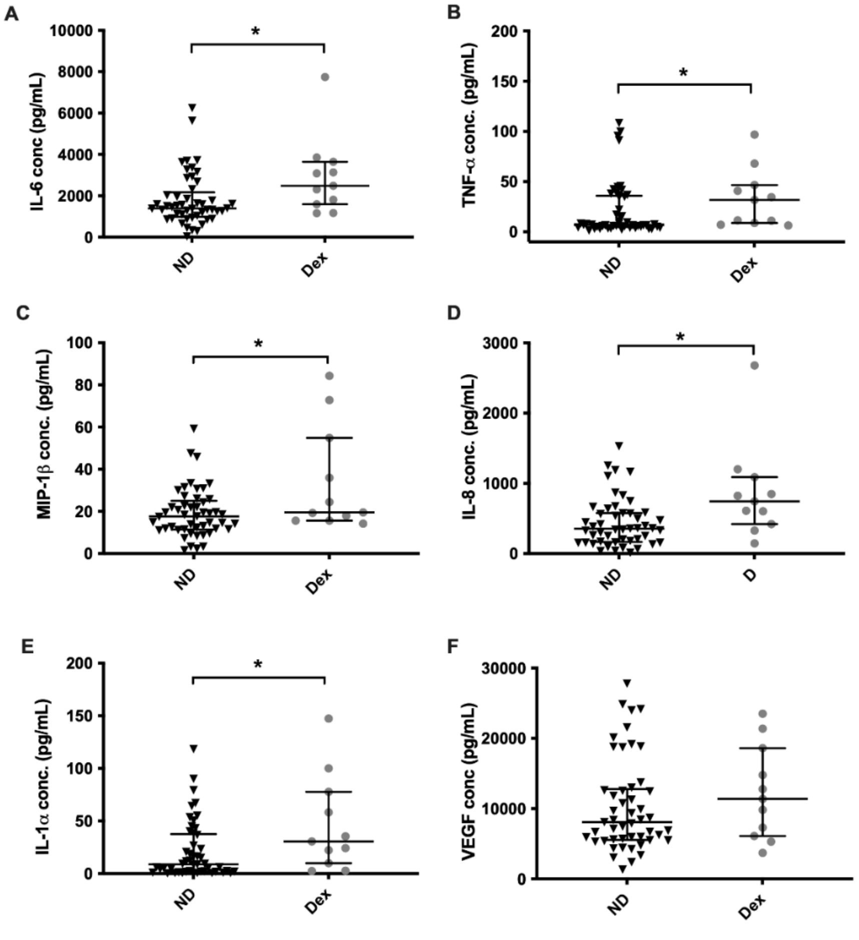

Figure S2; intra-operative inflammatory mediator concentrations in no-dexamethasone (ND) and dexamethasone (Dex) samples; **(A)** IL-6, **(B)** IL-8, **(C)** MIP-1β, **(D)** IL-1α, **(E)** TNF-α, **(F)** VEGF. ND = 49, Dex = 12. Line (median), bars (IQR), statistically significant differences denoted as $P \leq 0.05$ (*), $P \leq 0.005$ (**).

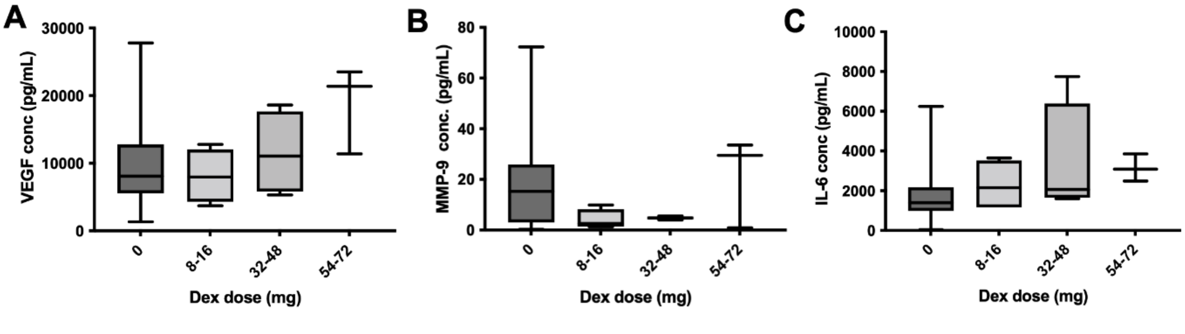

Figure S3; Intra-operative mediator concentrations in relation to cumulative pre-operative dexamethasone doses for; **(A)** VEGF, **(B)** MMP-9, **(C)** IL-6, with 0 mg (n = 49), 8-16 mg (n = 4), 32-48 mg (n = 4), 54-72 mg (n = 3)**.** Line (median), Box (interquartile range), whiskers (minimum-maximum), Dex = dexamethasone.

Table S1: Median mediator concentration in all primary CSDH samples comparing patients under 75 and 75 and older (NS = non-significant).

Mediator	Patients aged <75 (n=27)	Patients aged >74 (n=34)	P value (significance)
VEGF	8443	8881	0.857 (NS)
IL-6	1586	1512	0.512 (NS)
IL-8	419.3	371.8	0.790 (NS)
IL-10	26.23	34.76	0.269 (NS)
IL-1α	8.467	17.47	0.295 (NS)
IL-1β	1.529	1.052	0.217 (NS)
IP-10	286.4	234.8	0.236 (NS)
MCP-1	1519	1498	0.211 (NS)
MIP-1α	10.97	14.02	0.065 (NS)
MIP-1β	18.37	19.48	0.810 (NS)
MMP-9	13.79	12.89	0.639 (NS)
TNF-α	11.45	7.693	0.455 (NS)

Table S2: correlation of mediator concentrations in CSDH fluid between left and right sides of 10 bilateral CSDHs with Spearman correlation.

Correlation	Positive correlation between sides of bilateral CSDH
Strongly correlated	IL-1α, R = 0.930 ($p > 0.001$)<br>IL-1β, R = 0.748 ($p = 0.016$)<br>TNF-α, R = 0.891 ($p = 0.001$)<br>MCP-1, R = 0.879 ($p = 0.002$)<br>VEGF, R = 0.733 ($p = 0.020$)
Moderately correlated	IL-10, R = 0.636 ($p = 0.054$)<br>MIP-1β, R = 0.624 ($p = 0.060$)<br>IL-6, R = 0.515 ($p = 0.133$)<br>IP-10, R = 0.479 ($p = 0.166$)<br>MMP-9, R = 0.478 ($p = 0.166$)<br>MIP-1α, R = 0.418 ($p = 0.233$)
Poorly correlated	IL-8 , R = 0.139 ($p = 0.707$)
